# From *Hermetia illucens* Pupal Exuviae to Antimicrobial Composites: Metal Nanoparticles Synthesized by Laser Ablation in Sustainable Chitosan Matrices

**DOI:** 10.3390/molecules30163368

**Published:** 2025-08-13

**Authors:** Michela Marsico, Anna Guarnieri, Mariangela Curcio, Carmen Scieuzo, Roberto Teghil, Patrizia Falabella, Angela De Bonis

**Affiliations:** 1 Department of Basic and Applied Sciences, University of Basilicata, 85100 Potenza, Italy; michela.marsico@unibas.it (M.M.); anna.guarnieri@unibas.it (A.G.); curcio.mariangela@gmail.com (M.C.); carmen.scieuzo@unibas.it (C.S.); patrizia.falabella@unibas.it (P.F.); 2 Spinoff XFlies S.R.L, University of Basilicata, 85100 Potenza, Italy

**Keywords:** chitosan, *Hermetia illucens*, laser ablation, AgNPs, CuNPs, composite materials, antibacterial activity

## Abstract

Chitosan is a natural biopolymer with intrinsic antimicrobial properties and strong metal ion chelating properties, making it an ideal matrix for the development of bioactive composites. In this study, silver and copper nanoparticles were synthesized using laser ablation in liquid (LAL) by the ablation of metallic targets into commercial chitosan (Cs) and chitosan produced from *Hermetia illucens* pupal exuviae (CsE) solutions, avoiding the use of chemical precursors or stabilizing agents. The nanocomposites obtained were characterized by UV–vis spectroscopy, TEM microscopy and FTIR spectroscopy in order to evaluate the size of the nanoparticles and the interactions between the polymer and metal nanoparticles. Antibacterial tests demonstrated the efficacy of Ag-based composites with a minimum inhibitory concentration (MIC) of 0.006 g/L, and Cu-based composites with a MIC of 0.003 g/L against both *Escherichia coli* and *Micrococcus flavus*. While the silver composites show antibacterial activity in both colloidal and film forms, the copper composites present antibacterial activity only in colloidal form. Swelling tests indicated that all films maintained a high water absorption capacity, with a swelling index over 200%, unaffected by nanoparticle integration. The results highlight the potential of LAL-synthesized metal–chitosan composites, particularly those based on insect chitosan, as sustainable and effective antimicrobial materials for biomedical and environmental applications.

## 1. Introduction

Chitosan, obtained through the deacetylation of chitin, the second most abundant naturally occurring biopolymer after cellulose, has numerous properties, including biocompatibility, biodegradability, intrinsic antimicrobial activity and low production costs [1]. 

The growing market demand for this biopolymer has driven research into alternative and more sustainable sources than crustaceans [2]. One promising solution are bioconverter insects, particularly *Hermetia illucens* [3]. Pupal exuviae from its breeding represent a valuable biomass that is typically wasted, but that can offer chitin and chitosan yields, comparable to those obtained from the commercial source, making *H. illucens* a great biopolymer substitute [4].

Chitosan is widely used in biomedical and environmental fields for many applications such as being a carrier of active substances, wound healing, tissue engineering, water purification and controlled-release fertilizer [5,6]. The presence of amino and hydroxyl groups makes this biopolymer an excellent chelator and stabilizer for metal ions and nanoparticles [7].

This ability has triggered the research and production of metal–chitosan composites (Me-Cs), in which the biopolymer acts both as a stabilizing matrix and as a functionalising agent for metallic nanoparticles [8,9,10]. Such composites have proved particularly promising in applications such as heterogeneous catalysis, biosensors, the controlled release of drugs and particularly as biomaterials with enhanced antimicrobial capabilities [9,11]. The association between the antibacterial properties and the high film-forming capacity of chitosan gives metal–chitosan composites a wide application potential in the biomedical field; these materials are in fact suitable for use as functional coatings on fabrics with antimicrobial activity that can be used in hospital or daily use environments, as already demonstrated in previous studies on similar composites [12].

Numerous studies document the integration of chitosan with nanoparticles of noble and transition metals, such as gold (Au) [13,14], platinum (Pt) [15,16], palladium (Pd) [17,18], iron (Fe), copper (Cu) [19] and silver (Ag) [20]. 

These composites have found various applications in different fields: Au-Cs composites are widely explored for their usefulness in biosensing, controlled-targeted drug release systems and biomedical imaging due to their unique optical and electrical characteristics [13]. Pt-Cs nanocomposites are known to be effective in electrocatalysis [21], as advanced biosensors and for various biomedical applications, including anticancer agents [22]. Pd-CS materials are particularly important in heterogeneous catalysis [23,24] for environmental remediation through the degradation of pollutants [25]. Fe-Cs composites are widely studied for environmental decontamination by heavy metal removal or waste water treatment [26,27], magnetic resonance imaging (MRI) and nanomedicine for hyperthermia treatments [28,29]. Cu-Cs composites are useful in catalytic processes [30], as powerful antimicrobial [31] and antifungal agents [32] and in biosensors [33]. Finally, Ag-Cs composites are predominantly recognized for their powerful antibacterial, antiviral and antifungal activities [34,35,36], making them very promising in medical devices, wound healing and sensors [37]. Among these, Cu-Cs and Ag-Cs composites are of particular interest because of their synergistic antibacterial activity: the intrinsic antimicrobial properties of chitosan are enhanced thanks to the metal nanoparticles, making these materials potentially effective against a wide range of micro-organisms, including antibiotic-resistant strains.

Usually, such composites are obtained by chemical synthesis methods, often using metallic precursors in saline form (such as nitrates or chlorides) and reducing agents (such as borohydrides, ascorbic acid), which can leave unwanted residues on the surface of newly formed nanoparticles or require synthetic conditions that are not sustainable [38]. As an alternative to this, the technique of laser ablation in liquid (LAL) is emerging as a green, clean and versatile methodology for the synthesis of metal nanoparticles [39].

Liquid laser ablation consists of irradiating a metal target immersed in a liquid by high-energy laser pulses, generating nanoparticles directly in the solvent without the use of chemical precursors or stabilizers [40,41]. This method produces stable and highly reactive colloids, ready to be functionalized or incorporated into polymer matrices [39,42]. In addition, the modulation of laser parameters, wavelength, energy, pulse duration and frequency allows for the fine control of the morphological and optical properties of the nanoparticles produced, making the technique highly customizable [41,43].

In the present study, we propose the development of chitosan-based antimicrobial composites containing silver and copper nanoparticles obtained by LAL, a green synthesis methodology. Furthermore, in this research work, chitosan extracted from the pupal exuviae of *Hermetiaillucens* is used, which is still little valorised, and compared with commercial chitosan in the manufacture of metal–polymer nanocomposites. The specific objectives were to evaluate the possibility of using chitosan of entomological origin in the synthesis of nanoparticles via LAL, conduct a chemical–physical characterization of the composites obtained (UV–vis, TEM, FTIR), and lastly analyse and compare the antimicrobial efficacy both in colloidal and film form, which are relevant for applications in the biomedical and environmental fields. The use of a sustainable synthesis method together with the use of an unconventional biological resource at the same time promotes the principles of green chemistry and the circular economy.

## 2. Results and Discussion

### 2.1. Physico-Chemical Characterization

**Figure 1:**
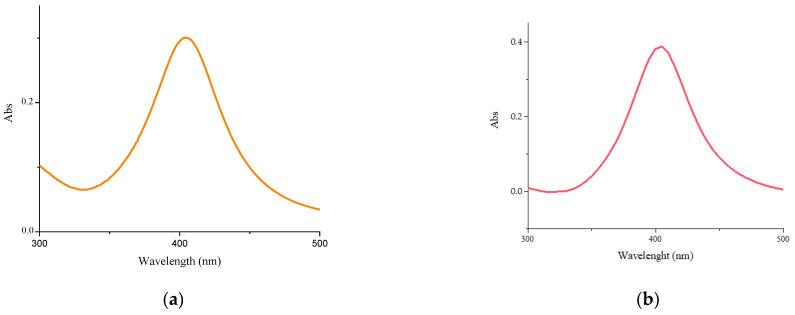

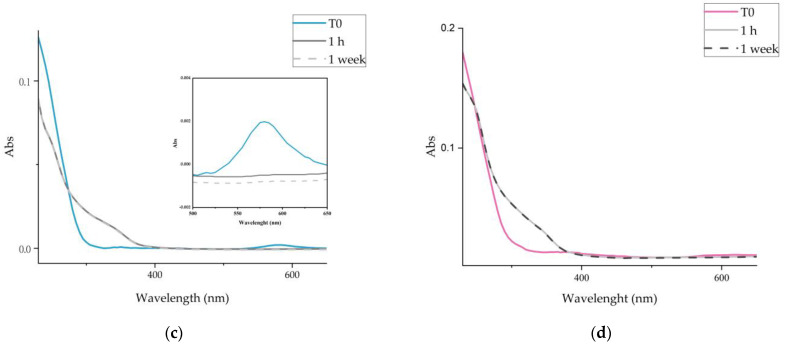
UV–visible spectra: (**a**) Cs-AgNPs, (**b**) CsE-AgNPs, (**c**) Cs-CuNPs, (**d**) CsE-CuNPs.

In Figure 1a,b the UV–vis absorbance spectra of the Cs-AgNPs and CsE-AgNPs are reported. Both spectra present a band at about 400 nm due to the surface plasmon resonance of silver nanoparticles [44], confirming the dispersion of metallic particles within the polymers. The AgNPs remain well dispersed in both polymeric matrices, as expected [45,46]**.**

The UV–vis absorption spectra of Cu-NPs synthesized in Cs and CsE solutions freshly prepared, visible in Figure 1c,d, show a band of very low intensity centred at about 580 nm, related to the plasmonic surface resonance of metallic copper. This band disappears rapidly (after 1 h from colloidal preparation) while two new shoulders appear, the first visible from 250 to 300 nm and the second from 300 to 400 nm, suggesting the formation of CuO and Cu_2_O respectively [47]. No further changes were observed in the following weeks. 

**Figure 2:**
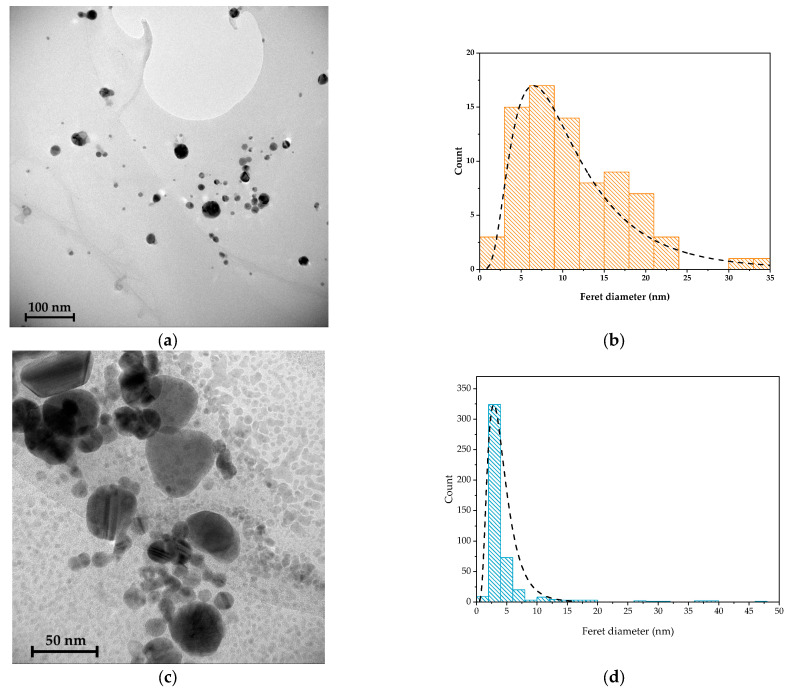
TEM images of (**a**) Cs-AgNPs and (**b**) Cs-AgNPs dimension distributions, and (**c**) Cs-CuNPs and (**d**) Cs-CuNPs dimension distributions.

The TEM image confirms the formation of AgNPs (Figure 2a,b) in the polymeric matrix. The metallic nanoparticles are spherical, with a mean diameter of 11 nm and are well distributed in the matrix. Unlike AgNPs, the size distribution of the Cu nanoparticles (Figure 2c,d) is characterized by a large amount of particles with a diameter between 5 and 10 nm and some larger particles, with edges in their shape. The coalescence of smaller nanoparticles is clearly visible in the TEM image (Figure 2c). 

**Figure 3:**
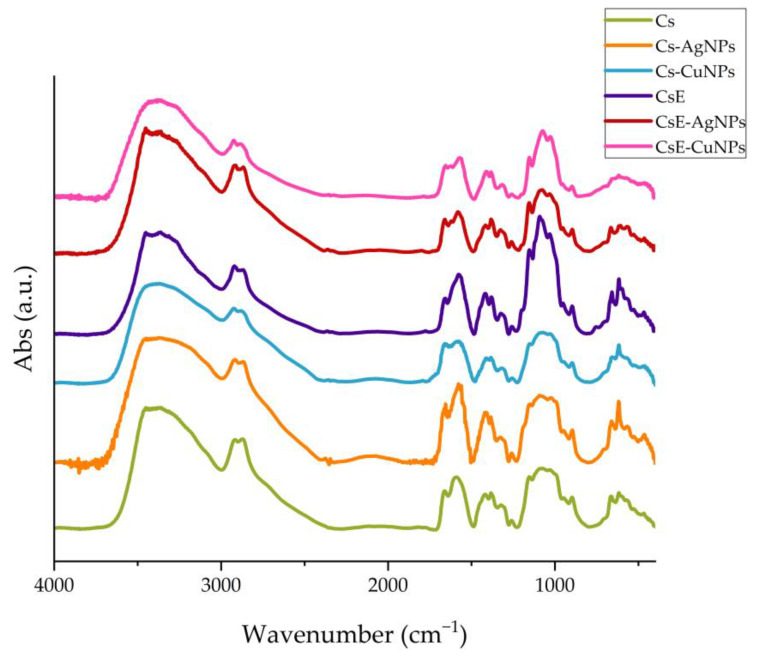
FTIR spectra: Cs (green), Cs-AgNPs (orange), Cs-CuNPs (light blue), CsE (purple), CsE-AgNPs (red) and CsE-CuNPs (pink) comparison.

The FTIR spectra of all samples are shown in Figure 3. The FTIR spectra of Cs and CsE show the characteristic bands of glucosamine-based polymers. In particular, a wide and intense band around 3400 cm^−1^ is observed, attributable to the stretching vibrations of the -OH and -NH groups involved in intra- and inter-molecular hydrogen bonds. Bands between 1650 and 1550 cm^−1^ are associated with the stretching vibrations of the C=O bond of the amide group and the deformation of the N-H bond [48]. At lower frequencies the vibrations of the carbon skeleton and other functional groups present in the structure of chitosan are detected. The FTIR spectra of the composites (Cs-AgNPs, CsE-AgNPs, Cs-CuNPs and CsE-CuNPs) confirm the preservation of the polymeric structure, as shown by the presence of typical bands, and at the same time show some variations in the region around 1570 cm^−1^, where there is a variation in the intensity and shape of the bands compared with the polymer [20,49]. These spectroscopic changes can be interpreted as an indication of the interaction between the amino groups of chitosan and the metal nanoparticles [50,51].

**Figure 4:**
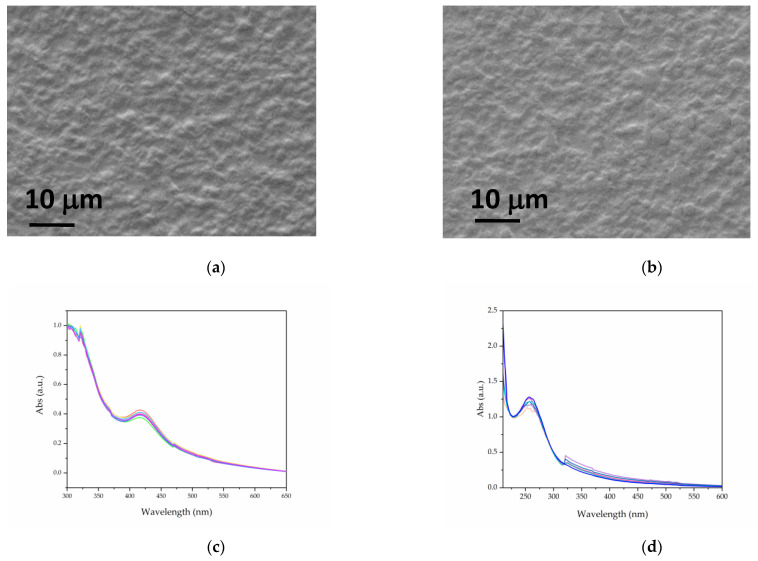
SEM images of Cs-AgNPs (**a**) and Cs-CuNPs (**b**). UV–visible spectra acquired at different points for Cs-AgNPs (**c**) and Cs-CuNPs (**d**) films.

The SEM images of the chitosan (CS) composites are shown in Figure 4a,b. The films exhibit a uniform, continuous, and slightly rough surface, which is characteristic of biopolymeric materials. The UV–visible spectra recorded at different points on the films (Figure 4c,d) reveal a variation of less than 15% in the surface plasmon resonance (SPR) absorbance of Ag (at 415 nm) and CuO (at 256 nm), indicating a relatively homogeneous distribution of the nanoparticles at the scale probed by this technique.

### 2.2. Antibacterial Tests

**Figure 5:**
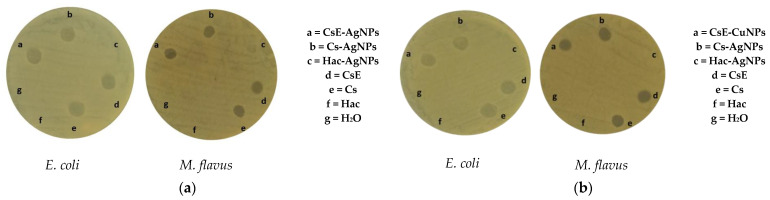
Inhibition zones of samples (**a**) AgNPs and composites and (**b**) CuNPs and composites on *E. coli* and *M. flavus* resulting from the agar diffusion test.

An evaluation of the antibacterial activity of the different synthesized compounds, Cs-AgNPs, CsE-AgNPs, Cs-CuNPs and CsE-CuNPs, was carried out on both *Escherichia coli* and *Micrococcus flavus*. The formation of inhibition zones around the composite samples is visible in the images reported in Figure 5, indicating a marked antimicrobial activity of these composites. Particularly, both compounds with AgNPs and those with CuNPs, in association with Cs or CsE, showed a comparable effectiveness in inhibiting the growth of both strains tested. In contrast, the negative control represented by sterile water did not induce any inhibition zones, as well as colloidal solutions of nanoparticles in acetic acid (Hac), Hac-AgNPs and Hac-CuNPs, which showed no detectable antibacterial activity. These results suggest that the observed antimicrobial efficacy is mainly due to the interaction between biopolymers (Cs and CsE) and metal nanoparticles. However, there was no statistically significant difference in antibacterial activity between the composite materials (CsE-AgNPs, Cs-AgNPs, CsE-CuNPs, Cs-CuNPs) and their base biopolymer matrices (Cs and CsE), showing a comparable effect in countering microbial proliferation. Quantitative data on the diameters of the inhibition zones are given in Table 1.

**Figure 6:**
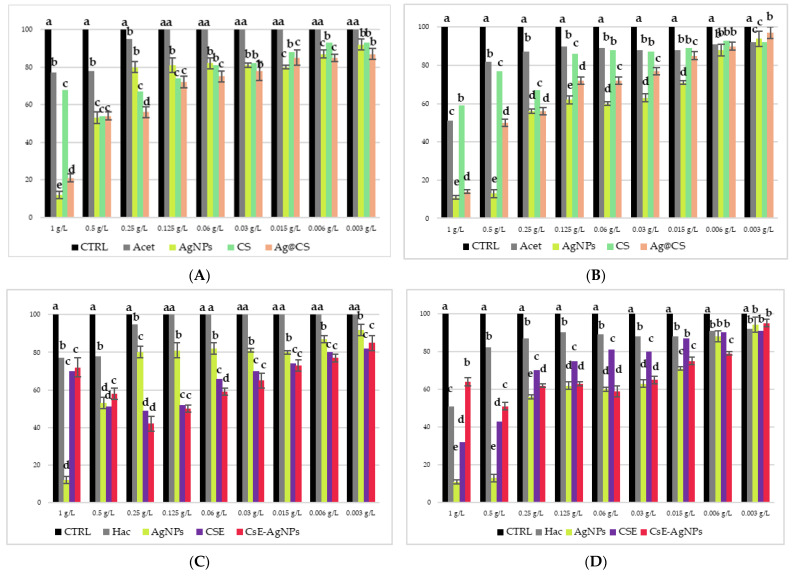
Results of microdilution assay for AgNPs combined with Cs, AgNPs combined with CsE, AgNPs in acetic acid, CsE, Cs, acetic acid and distilled water at the nine concentrations of 1, 0.5, 0.25, 0.125, 0.06, 0.03, 0.015, 0.006 and 0.003 g/L against *E. coli* (**A**,**C**) and *M. flavus* (**B**,**D**). Bars indicate the absorbance of the bacterial culture (black), the culture treated with Cs (green), CsE (purple), acetic acid (grey), AgNPs in acetic acid (yellow), Cs-AgNPs (orange) and CsE-AgNPs (red). Data are expressed as mean ± standard deviation. Different letters indicate significant differences (*p* < 0.05) between absorbance values of the bacterial culture alone and that of bacteria treated with the different concentrations of each treatment (data were analyzed with one-way Anova and Bonferroni’s *post hoc* test).

Figure 6 shows the results of the microdilution test performed on bacterial cultures of *E. coli* (Figure 6A,C) and *M. flavus* (Figure 6B,D) of the Cs-AgNPs and CsE-AgNPs samples. The analyses were conducted in a range of concentrations between 1 g/L and 0.003 g/L, including as controls only acetic acid solution (Hac), AgNPs in acidic solution and polymeric matrix solutions. At the highest concentration (1 g/L), all samples showed a significant inhibition of bacterial growth in both species, which is also attributable to the antimicrobial effect of acetic acid, known to be active at concentrations above 0.04% [52]. However, from 0.25 g/L and lower concentrations, this effect can be ruled out, as indicated by the Hac sample (grey bars), which shows negligible activity. In Figure 6A,B, referring to *E. coli*, it is clearly observed that the composite materials containing silver nanoparticles and chitosan show antibacterial activity, with a MIC determined at 0.006 g/L for both Cs-AgNPs and CsE-AgNPs composites. However, CsE-AgNPs are more effective in the whole range of concentrations from 0.5 to 0.006 g/L than both Cs-AgNPs and AgNPs in acid. This suggests a synergistic effect between chitosan derived from *H. illucens* and AgNPs. Materials containing only the biopolymer CS and CsE show less antimicrobial activity, while maintaining a consistent trend between them. For *M. flavus* (Figure 6B,D), the antimicrobial activity of CsE-AgNPs and Cs-AgNPs is overall lower than that observed on *E. coli*, with less marked differences compared with AgNPs in acid solution. AgNPs in acid show at higher concentrations (1–0.25 g/L) a large inhibitory effect, with bacterial growth less than 50% when compared with the control. However, even in this case, the MIC for CsE-AgNPs and Cs-AgNPs turns out to be 0.006 g/L, indicating the substantial effectiveness of the composites also against Gram-positive strains. Chitosan matrices alone (CsE and Cs) show a comparable effect only at lower concentrations (0.015–0.003 g/L), while at concentrations from 0.5 to 0.06 g/L, LCsE is more effective than Cs.

**Figure 7:**
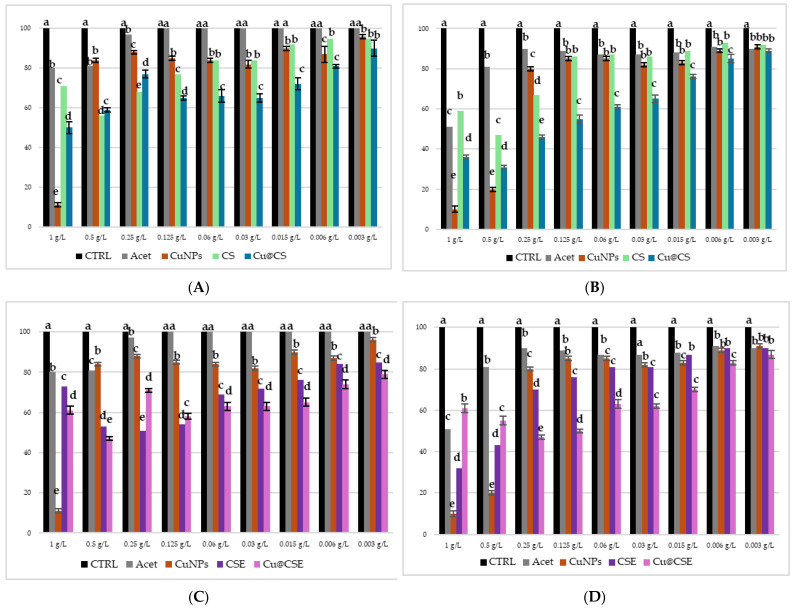
Results of microdilution assay for CuNPs combined with Cs, CuNPs combined with CsE, AgNPs in acetic acid, CsE, Cs, acetic acid and distilled water at the nine concentrations of 1, 0.5, 0.25, 0.125, 0.06, 0.03, 0.015, 0.006 and 0.003 g/L against *E. coli* (**A**,**C**) and *M. flavus* (**B**,**D**). Bars indicate the absorbance of the bacterial culture (black), the culture treated with Cs (green), CsE (purple), acetic acid (grey), CuNPs in acetic acid (dark red), Cs-CuNPs (blue) and CsE-CuNPs (violet). Data are expressed as mean ± standard deviation. Different letters indicate significant differences (*p* < 0.05) between absorbance values of the bacterial culture alone and that of bacteria treated with the different concentrations of each treatment (data were analyzed with one-way Anova and Bonferroni’s *post hoc* test).

Figure 7 shows the results of the microdilution test conducted on *E. coli* (Figure 7A,C) and *M. flavus* (Figure 7B,D) at different concentrations of Cs-CuNPs and CsE-CuNPs composite material, at their respective biopolymer matrix concentrations; also in this case the acetic acid solution and CuNPs in acidic solution were evaluated as controls. The solutions were tested in a range of concentrations between 1 g/L and 0.003 g/L. At the two highest concentrations (1 and 0.5 g/L), all tested samples showed a marked inhibitory effect against both bacterial strains, an effect partly attributable, as already mentioned, to the antimicrobial activity of acetic acid, whose effect however, from 0.25 g/L and below can be considered negligible, as confirmed by the experimental data shown in Figure 6. In relation to *E. coli* (Figure 7A,C), the composite materials CsE-CuNPs and Cs-CuNPs demonstrated significantly higher antimicrobial activity than the polymer matrices and nanoparticles in acid solution. Particularly, CsE-CuNPs showed inhibition capacities, with a MIC identified at 0.003 g/L, which was also replicated by Cs-CuNPs, albeit with a slightly lower activity. These results suggest a synergistic effect between copper nanoparticles and chitosan matrices, which are more pronounced when using chitosan extracted from *H. illucens*. In line with this, CsE shows a higher antibacterial activity than commercial CS in the whole range of concentrations below 0.5 g/L, indicating the efficacy of chitosan derived from pupal exuviae. In the case of *M. flavus* (Figure 7B,D), CsE-CuNPs and Cs-CuNPs composites show inhibitory activity, especially at low concentrations. Also in this case, it is observed that the MIC for both composite materials is detectable at 0.003 g/L, while the individual components (Cs, CsE, CuNPs) are less effective. The colloidal material product showed significant activity against *E. coli* and *M. flavus*, in line with what was reported for similar composite materials, in various studies also carried out on other bacterial strains [53,54,55,56].

**Figure 8:**
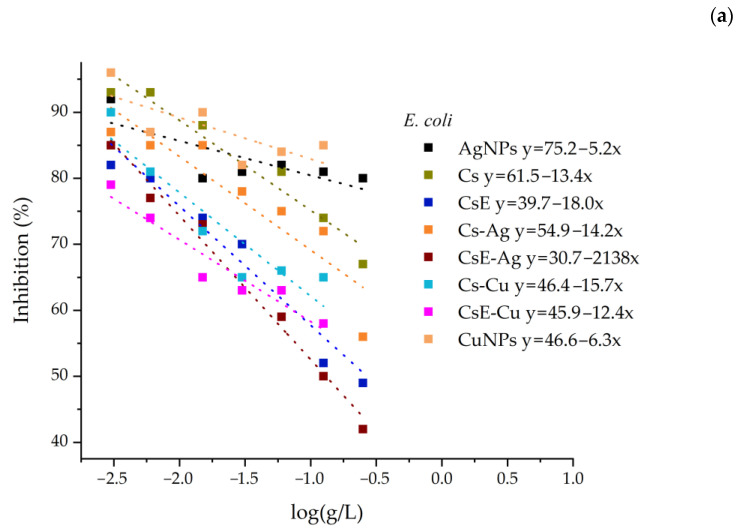

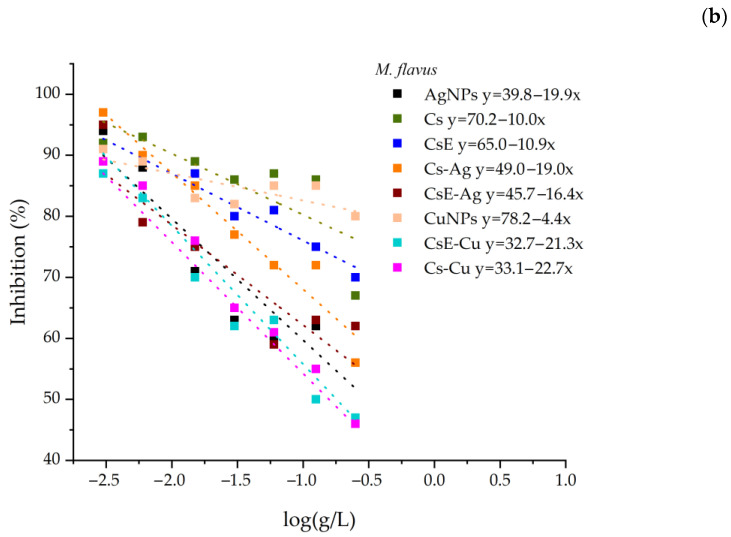
Inhibition of all samples against *E. coli* (**a**) and *M. flavus* (**b**). The linear fitting equations are shown in the legend.

The IC50 values for the inhibition against *E. coli* and *M. flavus* for all samples were calculated by the linear fit of the OD measurements (Figure 8). However, these data occasionally show some degree of non-linearity, particularly at higher concentrations. This effect is primarily attributable to the characteristics of chitosan, which, at high concentrations, can form solutions with an opalescent or turbid appearance. Moreover, the presence of insoluble or semi-crystalline residues may remain suspended in solution and interfere with the measurements. Insect-derived chitosan may also contain residual impurities, such as pigments and proteins, which could potentially contribute to altered spectrophotometric measurements. Considering these effects, the IC50 values of all samples were calculated by excluding the data points at higher concentrations (Table 2).

**Figure 9:**
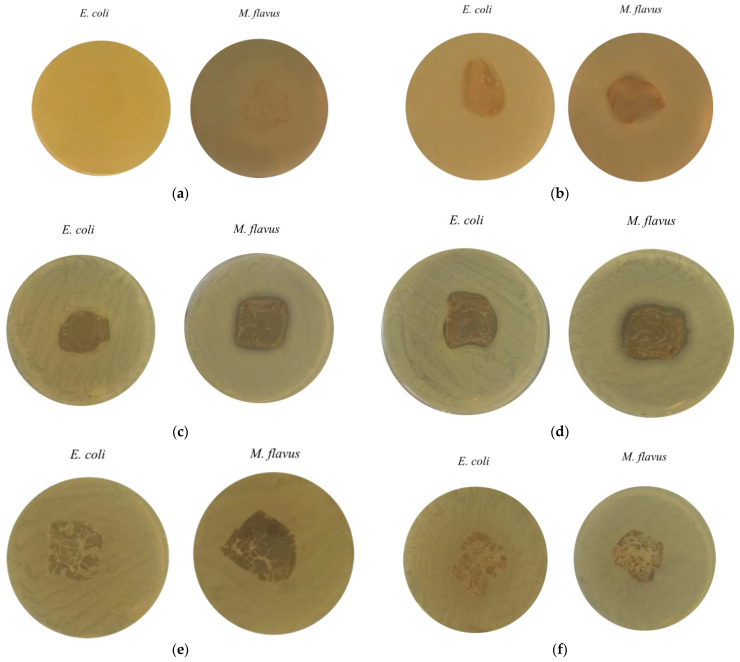
Inhibition zones from the agar diffusion test on *E. coli* and *M. flavus* films: (**a**) Cs; (**b**) CsE; (**c**) Cs-Ag; (**d**) CsE-Ag; (**e**) Cs-Cu and (**f**) CsE-Cu.

Figure 9 shows the results of the agar diffusion test conducted to assess the antibacterial activity of chitosan-based composite films, with or without metal nanoparticles, applied on the two bacterial strains (*E. coli* and *M. flavus*). The samples are labelled with letters (a) to (f), corresponding to the following materials: (a) commercial chitosan (Cs), (b) pupal exuviae (CsE)-derived chitosan, (c) Cs-Ag, (d) CsE-Ag, (e) Cs-Cu, (f) CsE-Cu. In Figure 9a, concerning Cs films, there are no clear inhibition zones around the film on both Gram-negative and Gram-positive bacteria, suggesting a very weak antimicrobial activity of chitosan in solid form. In Figure 9b, where the CsE film inhibition zone is shown, a similar effect can be observed as seen in the case of Cs films. Figure 9c and d show the results obtained, respectively, with Cs and CsE combined with AgNPs. In both cases, well defined inhibition zones are observed around the films for both bacterial strains. These results indicate significant antibacterial activity conferred by the presence of AgNPs, consistent with the well known mechanism of release of Ag ions and consequent interference with vital cellular processes [57]. Slight differences are observed between Cs-Ag(c) and CsE-Ag (d), with the presence of more extensive and evident inhibition zones on both strains, in the case of the composite obtained from the CsE matrix. Figure 9e,f, containing, respectively, Cs and CsE films in combinations with CuNPs, do not show the inhibition zones. It is possible to observe the formation of colonies close to the surface of the films; therefore, a lack of antibacterial activity is indicated, contrary to expectations and the antithesis observed for counterparts enriched with silver nanoparticles. These results indicate that while silver films retain significant antibacterial activity in solid form, copper composites may lose their effectiveness in film form, potentially due to reduced ion release or the surface oxidation of nanoparticles. These observations are consistent with reports in the literature, where chitosan–copper composite films prepared under similar conditions did not exhibit antibacterial activity in solid-state tests against *S. aureus* [58]. Several studies have shown that, to achieve a measurable bacteriostatic or bactericidal effect, copper must be present at concentrations above a minimum threshold. Specifically, minimum inhibitory concentration (MIC) values of approximately 22 μg/mL have been reported [59]. This suggests that the copper content in the films analyzed in this study may fall below this threshold, thereby limiting the antimicrobial efficacy of the material.

All results for the antibacterial activity is resumed in Table 3. 

**Figure 10:**
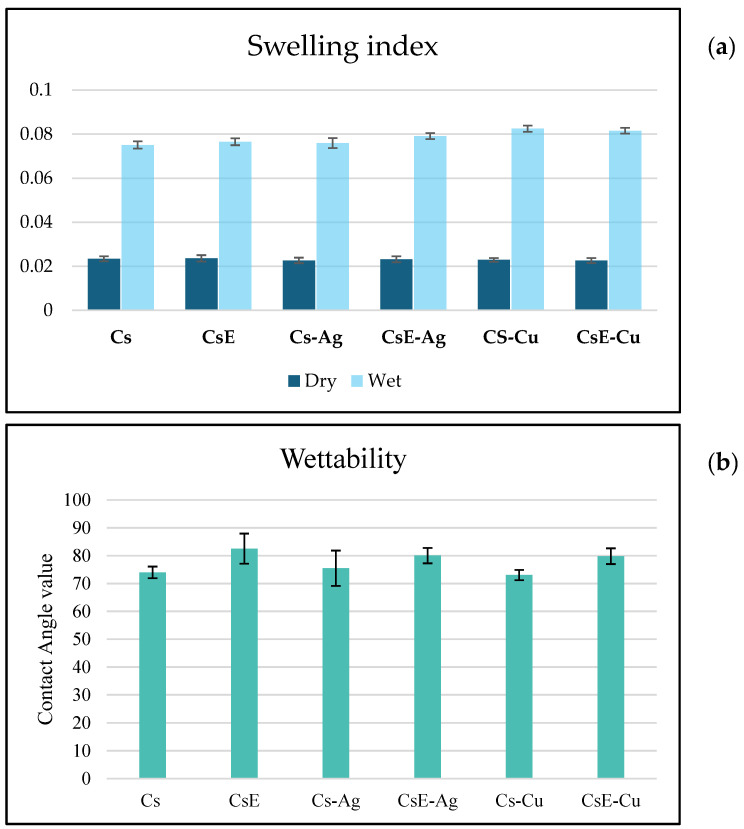
(**a**): Swelling index of the films are shown as average values, the weights of the dry films and the average weights of the films after immersion in water for 2 h. Data are expressed as mean ± SD. (**b**) Contact angle measurements. Data are expressed as mean ± SD.

From the data shown in Figure 10, concerning the swelling index in water, the films analyzed showed a high capacity of water absorption, with an average dry weight of about 0.023 g and an average wet weight between 0.075 g and 0.085 g, corresponding to a swelling index of more than 200%. This behaviour confirms a marked affinity of the materials for water. These findings are supported by wettability measurements (Figure 9b). The contact angle measured ranges in the range of 72–82° for all the samples. It is interesting to note that the different types of commercial chitosan (Cs), exuviae chitosan (CsE) films and their related composites Cs-Ag, Cs-Cu, CsE-Ag and CsE-Cu present very similar bulging values, with slight variations. Particularly, the insertion of metal nanoparticles does not appear to significantly impair the polymer ability to absorb water, suggesting that the matrix interactions with metal nanoparticles do not interfere with the interactions between chitosan functional groups and water molecules. In addition, CsE showed a behaviour almost identical to that of Cs. This is of relevance for applications in the biomedical and environmental fields, as it suggests that materials retain good hydrophilicity even after integration with metal nanoparticles.

## 3. Materials and Methods

### 3.1. Materials

Chitosan (Acros, molecular weight 100,000–300,000 Da and with 70% deacetylation degree), chitosan from pupal exuviae of *H. illucens* (molecular weight about 35,000 Da and with 75% deacetylation degree, provided from Xflies s.r.l (Potenza, Italy)) [4], acetic acid (99.8%) and sodium hydroxide reagents were acquired from Sigma-Aldrich (Saint Louis, MO, USA). High-purity copper metal (Cu ≤ 99.999%) and high-purity silver metal (Ag ≤ 99.999%) were purchased from Goodfellow (Cambridge, UK).

### 3.2. Preparation of Chitosan Solutions 

Firstly, 1g/L of chitosan (Cs) solution was prepared by dissolving it in 0.1% acetic acid, at a pH of 4.2. The solution was stirred at 35 °C for 4 h until complete dissolution. The same process has been adapted to obtain pupal exuviae chitosan (CsE) solution (1 g/L).

### 3.3. AgNPs and CuNPs Synthesis

Laser ablation experiments were performed by using a Nd:YAG (Handy-YAG-Quanta System) (Quanta System, Milano, Italy) laser source operating at 532 nm wavelength, repetition rate of 10 Hz, pulse duration of 7 nm and power of 150 mW. The laser source was focused vertically on the surface of the solid target, silver or copper, by means of a 50 mm focusing lens and the target was immersed in 50 mL of polymer solution. The concentrations of nanoparticles obtained were evaluated by considering the mass of metal targets before and after ablation. The solutions prepared are reported in Table 4.

### 3.4. Preparation of Films

The colloidal solutions were dropped into vessels (3.5 × 3.5 cm) and dried at 25 °C until the solvent completely evaporated. The resulting films were neutralized with sodium hydroxide (NaOH) 0.05 M solution and then rinsed in distilled water to complete neutrality. Finally, the films were left to dry at room temperature.

### 3.5. Physico-Chemical Characterization 

The optical properties of the produced colloidal solutions were investigated by UV–visible spectroscopy, and absorption spectra were acquired using a UV–vis spectrophotometer (model Specord 50/PLUS, Analytik Jena, Jena, Germany), operating in the spectral range between 200 and 800 nm with a resolution of 0.2 nm. The optical absorbance of the films was measured in 10 positions for an area of 2 × 2 mm by using the same UV–vis spectrophotometer. 

The TEM analysis allowed the study of the morphology, size distribution and crystal structure of the synthesized nanoparticles. The colloidal solutions were deposited on carbon film-coated copper grids (Agar Scientific) and then allowed to dry at room temperature. They were subsequently analyzed using a G2 20 FEI Tecnaitransmission electron microscope (FEI, Hillsboro, OR, US) operating at an acceleration voltage of 200 kV.

Some drops of colloidal solutions were deposited on monocrystalline silicon wafers (Si (100), Goodfellow, Cambridge, UK). After evaporation of the solvent, the samples thus prepared were analyzed directly by Fourier transform infrared spectroscopy (FTIR), using an uncoated silicon wafer as a reference. The FTIR spectroscopic analysis was carried out by the Jasco J-460 spectrophotometer (Jasco, Halifax, NS, Canada); absorption spectra were acquired in the spectral range between 4000 and 400 cm^−1^.

A scanning electron microscope (SEM Carl Zeiss Auriga with EDS, X-MaxN Oxford Instruments, Oberkochen, Germany) was used to study the surface morphology of the produced samples. Prior to SEM analysis, the films were coated with gold (Q150/S, Quorum Technologies™, Lewes, UK) to avoid the charging effect on the sample

To evaluate the swelling index, the films were immersed in distilled water at room temperature (24 °C) until equilibrium (24 h). The weight of the wet samples (*Ws*) was measured after removing the surface water with absorbent paper. The swelling index (*Si*) was then calculated based on the weight of wet (*Ws*) and dry films (*Wd*) using the following equation.

(1) $$Si=\left(\frac{Ws-Wd}{Wd}\right)\times 100$$

The wetting properties were evaluated by contact angle measurement for each film: a drop of distilled water with a volume of 3 μL was dropped onto the centre of each sample to avoid any effects from the edges, and operation was repeated three times for each sample. The mean value and standard deviation of the contact angle identified were then calculated.

### 3.6. Antibacterial Tests

Bacterial colonies of *Escherichia coli* (Gram-negative) and *Micrococcus flavus* (Gram-positive) were inoculated in sterile culture medium. Specifically, bacteria, one colony per strain, were inoculated into sterile Luria–Bertani (LB) medium. The medium contained 0.5% sodium chloride (Sigma-Aldrich, St. Louis, MO, USA), 0.5% of yeast extract (Sigma-Aldrich, St. Louis, MO, USA) and 1% of tryptone (Sigma-Aldrich, St. Louis, MO, USA). The cultures were then incubated in a water bath shaker at 37 °C and 150 rpm for 18 h, in order to ensure bacterial growth.

### 3.7. Agar Diffusion Test

The antimicrobial activity of the synthesized composites was evaluated using the agar diffusion test; *E. coli* and *M. flavus* bacteria were distributed on solidified LB-Agar (LB with 1.5% bacteriological agar from Sigma-Aldrich, St. Louis, MO, USA). Subsequently, each colloidal solution was applied. For each plate, acetic acid, Cs solution and CsE solution at the same concentrations were used as positive controls, while sterile water was used as the negative control. The diameter of the inhibition zones (mm) is indicative of antimicrobial activity. The results were reported as averages and standard deviations of three independent biological replicates.

The antimicrobial activity of the film-like composite material was assessed by agar diffusion test. Bacteria were inoculated separately in LB culture medium (Sigma-Aldrich, St. Louis, MO, USA) enriched with agar (1.5% *w*/*w*) and homogenized. The plates were incubated at 37 °C for 24 h. Antimicrobial activity was quantified by measuring the inhibition zone around the films.

### 3.8. Minimum Inhibitory Concentration (MIC) by Microdilution Assay

Serial dilutions of all synthesized samples were prepared in 96-well plates, at the following concentrations: 1, 0.5, 0.25, 0.125, 0.06, 0.03, 0.0015, 0.006 and 0.003 g/L. Acetic acid solutions were also tested at equivalent concentrations (0.1, 0.05, 0.025, 0.0125, 0.006, 0.003, 0.0015, 0.0006 and 0.0003%, respectively), and each bacterial culture (*E. coli* and *M. flavus*) was employed as control. Bacterial cultures were used at a concentration of 10^6^ CFU/mL for both species. The 96-well plates were incubated at 37 °C for 24 h and bacterial concentrations were evaluated by measuring the optical density (OD) at 600 nm using a Multiskan Go spectrophotometer (Thermo Scientific, Waltham, MA, USA). The minimum inhibitory concentration (MIC) was defined as the lowest concentration of chitosan at which no bacterial growth was observed.

### 3.9. Statistics

Statistical analyses were carried out using Prism v. 5.0 (GraphPad Software Inc., La Jolla, CA, USA). Data were expressed as mean ± SD. Multiple comparisons were based on one-way or two-way analysis of variance (ANOVA), with Bonferroni’s *post hoc* test (*p* < 0.05).

## 4. Conclusions

Silver and copper nanoparticles were synthesized via nanosecond laser ablation in diluted polymeric solutions using both commercial chitosan (Cs) and chitosan extracted from the pupal exoskeletons of *Hermetia illucens* (CsE). Physico-chemical characterizations confirmed the formation of metallic nanoparticles and the preservation of the characteristic functionalities of the chitosan matrices. The antibacterial activity of the synthesized nanocomposites was evaluated against *Escherichia coli* (Gram-negative) and *Micrococcus flavus* (Gram-positive) using agar diffusion and microdilution assays. Both Cs and CsE enriched with silver nanoparticles exhibited notable antibacterial properties, with a minimum inhibitory concentration (MIC) of 0.006 g/L. Composites containing copper nanoparticles showed even lower MIC values of 0.003 g/L against both bacterial strains, indicating strong antibacterial efficacy in solution. A clear synergistic effect between the metal nanoparticles and the chitosan matrix was observed, particularly when using CsE. However, when processed into films, only silver-based materials retained substantial antibacterial activity, while copper-based films largely lost their efficacy. This suggests potential differences in metal ion release or nanoparticle stability within the solid-state matrix. Swelling index analysis further demonstrated that the incorporation of nanoparticles did not significantly affect the intrinsic water absorption capacity of the chitosan polymers. In conclusion, this study demonstrates the potential of laser ablation-synthesized chitosan–metal nanocomposites, especially those derived from sustainable insect-based chitosan sources, as promising antimicrobial materials. Their strong antibacterial activity, combined with environmentally friendly synthesis, supports their use in biomedical and environmental applications such as advanced biomaterials and water purification systems.

## Figures and Tables

**Table 1:** Diameters (mm) of inhibition zones formed by samples on *E. coli* and *M. flavus*. Distilled water was tested as negative control. Results are expressed as mean ± standard deviation of diameters measured with agar diffusion test of three independent biological replicates.

Sample	*E. coli*	*M. flavus*
Distilled water	/	/
Acetic acid (Hac)	/	/
Cs	8.5 ± 0.4	8 ± 0.3
CsE	8 ± 0.2	8.5 ± 0.2
AgNPs-Hac	/	/
Cs-AgNPs	8 ± 0.5	9 ± 0.5
CsE-AgNPs	9 ± 0.4	10 ± 0.4
CuNPs-Hac	/	/
Cs-CuNPs	8 ± 0.4	8.5 ± 0.4
CsE-CuNPs	7 ± 0.4	7 ± 0.4

**Table 2:** Minimum inhibitory concentration (MIC) and IC50 of the samples tested by microdilution assay on Gram-positive *M. flavus* and Gram-negative *E. coli*.

	*E. coli*		*M. flavus*	
Sample	MIC	IC 50	MIC	IC 50
Cs	0.015 g/L	7.0 g/L	0.030 g/L	107 g/L
CsE	0.015 g/L	0.27 g/L	0.015 g/L	23 g/L
AgNPs-Hac	0.015 g/L	67.0 g/L	0.015 g/L	0.31 g/L
Cs-AgNPs	0.006 g/L	2.24 g/L	0.006 g/L	0.88 g/L
CsE-AgNPs	0.006 g/L	0.13 g/L	0.006 g/L	0.55 g/L
CuNPs-Hac	0.006 g/L	>1000 g/L	0.015 g/L	>1000 g/L
Cs-CuNPs	0.003 g/L	0.59 g/L	0.003 g/L	0.18 g/L
CsE-CuNPs	0.003 g/L	0.47 g/L	0.003 g/L	0.16 g/L

**Table 3:** List of the samples analyzed, both in liquid and solid form. The antibacterial activity is indicated by "x".

	*E. coli*		*M. flavus*	
Sample	Sol	Film	Sol	Film
Cs	x		x	
CsE	x		x	
Cs-AgNPs	x	x	x	x
CsE-AgNPs	x	x	x	x
Cs-CuNPs	x		x	
CsE-CuNPs	x		x	

**Table 4:** Composition of the tested samples and its assigned name.

Sample	Polymer	MeNps
Cs	1 g/L Chitosan	/
CsE	1 g/L Pupal exuviae chitosan	/
Cs-AgNPs	1 g/L Chitosan	8 μg/mL
CsE-AgNPs	1 g/L Pupal exuviae chitosan	8 μg/mL
Cs-CuNPs	1 g/L Chitosan	15 μg/mL
CsE-CuNPs	1 g/L Pupal exuviae chitosan	11 μg/mL

## Data Availability

All data are available upon request.
